# Robinson’s Fibrous and Textile Filling

**Published:** 1884-06

**Authors:** 


### CORRESPONDENCE.

### Robinson's Fibrous and Textile Filling.

GRAND RAPIDS, MICH., May 1, 1884.

EDITOR DENTAL REGISTER: Having read in the March number of the *Ohio State Journal of Dentistry* an article by Dr. Brophy, of Chicago, relative to the merits of the fibrous and textile filling invented by Dr. J. A. Robinson, of Jackson, Mich., and, believing it worthy of commendation from others in the dental profession, I take this opportunity of penning my experience with the metal as a filling for teeth, and also as to its use as a lining for rubber plates. I have used this material quite extensively for more than two years, and feel that it is as indispensable as any other one material in use. I would not think it possible to do the best work without it. When used with gold as a filling it is *better* than gold alone, especially in proximal cavities in bicuspid and molar teeth, which extend close up to and above the margin of the gums. My way of using it in these cases is to place a piece of the fibrous and textile filling large enough to be tightly wedged into the cavity, so it will not rock and fill it at the cervical margin by a lap over the edge; pack it firmly with the mallet. I use Varney's points, then fill the body of cavity and finish with gold. By being particular and careful it is very easy to make an absolutely tight filling at the margin of the gums, and when finished have a beautiful filling, one that will not be nearly as apt to fail as when gold alone is used. I find no trouble in making gold unite to it, and I use this material in nearly every instance in the place of amalgam, believing it to be a better way to save the teeth, and nearly as easily used as amalgam. As far as gaining a reputation for filling teeth is concerned it pays to use it in preference to amalgam, as when your patient leaves the office you know what you have, whereas with amalgam a you only surmise that you have a good filling, and that only for short time; for with amalgam (you can only depend on it for a year or two at the longest) your plug is leaking, and your patient says: "No more filled teeth for me; I had them all filled only a short time ago, and now I want a new set." I had just such an experience to-day as the above, and have had many like cases. No doubt every dentist comes in contact with similar ones. Have asked several agents if they carried it. They say yes; but do not urge the sale of it, as it would interfere with the sale of amalgams, and there is a much greater profit on amalgams than on this. These special friends to the welfare of the dentists keep this article packed away in their trunks only to be brought out when called for. I hope every dentist, who looks out for the welfare of his patient, as also his own welfare—so far as his reputation for lasting work is concerned—will give this material a thorough trial, and am confident he will find it impossible to get along without it. I have used it with great satisfaction to myself and my patients for lining plates; have no trouble in putting it on, and simply follow the directions that come with it. Am glad to see this subject being brought forward, and hope it will be continued to be agitated until every dentist has the courage to give it a fair trial. A. R.

